# Anchorage of Gold Fillings

**Published:** 1893-02

**Authors:** S. B. Palmer

**Affiliations:** Syracuse, N. Y.


ARTICLE III.

### ANCHORAGE OF GOLD FILLINGS.

BY S. B. PALMER, M. D. S., SYRACUSE, N. Y.

Read at the Twenty-fourth Annual Union Dental Convention of the Sixth, Seventh and Eighth District Dental Societies of the State of New York, held at Binghamton, October 25th, 26th and 27th, 1892.

In rearing structures of importance, whether bridge or building, a good foundation should be the first consideration. This principle applies as well to the foundation of fillings in teeth, which rest upon dentine as different in structure and durability as rock and sand. It is customary to speak of this subject as anchorage, while foundations would better express the conditions The architect's ideal foundation is "solid rock," yet he must contend with all the intermediate grades to "sinking sand."

To obtain good results from filling, it is as important that the structure and condition of the dentine be understood, as how to insert a gold filling. There must be harmony between the two or failure will be the result. At least three conditions are necessary to produce permanent gold fillings: normal dentine, accessible cavities, and good manipulation.

No 1.—The ideal filling is one of cohesive gold throughout, well contoured and anchored in firm tooth structure. These happy combinations are not always present, and the requirements must be met by combinations of other preparations. It is true that a few skillful operators claim to accomplish all that is required with cohesive gold alone. Allowing this claim, there is a waste of energy for the operator and needless strain upon the patient, without corresponding benefits. One thing should not be lost sight of: that it is a fixed law in nature that a metal filling in the mouth exerts an electro-potential influence to decompose the fluids that may be between the filling and the walls of the cavity, or in the dentine itself, when that is much below normal density. This decomposition is according to the conductivity of the plug and its powers to resist oxydation. Thus, gold being the best conductor and free from oxydation, it acts with greater persistency than tin or amalgam. Let no one be deceived, and make a mistake in trying to circumvent this law by perfect manipulation, without a cavity lining which will exclude moisture. Although cavity lining is not anchorage, it has much to do in laying the foundation for fillings. Your attention is called to a few specific cavities and conditions of dentine, with recommendations for treatment in starting fillings:

First. Assuming that an accessible cavity is properly prepared in dentine of normal structure, with undercuts or angles so that the first piece of gold inserted is held firmly, upon which every other piece is made to cohere until the plug is completed, no finer filling can be produced, in appearance, or for tooth preservation, and thus credit is given to all who can do this superior work.

Care should be taken not to drill deep anchor pits into the vital dentine, as formerly practiced. Much harm has been done by or through the thermal changes thus introduced.

No. 2.—Foundations of soft gold for cohesive plugs.

A large proportion of gold fillings are made in this manner, and probably each operator has a method which suits him well. The one which best answers my purpose is to commence with one or more cylinders. Without describing the kind of cylinder recommended, we might as well say pellets. There is nothing in the market that answers the purpose, and we may better give a description how to make cylinders now, than later on.

Ney's No. 4 foil is best adapted for cylinders, on account of the roughness imparted to it by the book paper under pressure. Smooth foil makes cylinders too hard. The leaf may be cut into half, third or one-fourth strips, the ribbons folded to a width according to the depth of the cavity. Cylinders should be kept on hand and the selection made as required. The narrow strips are rolled around a broach, or better, a three-sided point attached to a firm handle. When the roll is drawn from the point, the ends should be slightly pressed with the pliers.

Let us now consider a cavity located in the grinding surface of molars, medium or large, surrounded by firm enamel borders, the bottom of the cavity flat or concave, as the case may be on the removal of the decalcified dentine. If the dentine is firm and dense, the cavity is ready for gold. When soft and sensitive, yet firm enough to warrant filling, varnish the cavity with some quick drying varnish. I am using Canada balsam cut in chloroform. Without waiting for the varnish to harden, line the bottom of the cavity with a piece of gold foil of two or three thickness. Upon this lining place a cylinder which will about fill the orifice of the cavity; if too large, compress it. The length of the cylinder may be one-half or two-thirds the depth of the cavity. Then commence with cohesive gold. Every piece introduced remains firm and is driven into the folds of the cylinder, condensing and expanding laterally every portion of the plug at the base, giving lateral pressure against the walls of the cavity, and the anchorage is perfect.

Condition No. 3.—Approximate cavities in molars or bicuspids, extending from the cervical border to the grinding surface.

Fillings contoured. Matrices well adapted to this class of work are made of rolled alluminum, wide enough to extend from the gum to one-half the length of the crown, and beveled to a knife edge. Insert a matrix of suitable thickness and bend the ends away from the tooth, thus giving full view and access to the cavity. The matrix is used only to prevent the first pieces of gold from falling out, and not to mould the filling. The cavity being in readiness, the portion next to the gums only should be varnished, as any like coating on the enamel, or even the dentine, prevents the mechanical bite which gold has without an enamel varnish.

Select a cylinder smaller than the cavity, but longer than its depth. Carry one end of the cylinder into the cavity, and force the outer end towards the gums upon the incline made by the knife-edge matrix. When the cylinder has been firmly pressed to the cervical border of the cavity, another cylinder may be placed by its side, or one on either side, and packed as before. Thus a firm and broad foundation is laid upon which cohesive gold may be packed to finish. When done, one end of the albuminum is bent straight, and the piece drawn out laterally. When the projecting ends of the cylinders are condensed and the plug finished, no portion of the filling will be more perfect than that at the cervical border.

Condition No. 4.—Same as the last described, except that the cavity extends beneath the gums. A case in practice will be sufficient.

A patient, while absent, had occasion to have a bicuspid filled. The cavity extended beneath the gums. The dam was applied and two unsuccessful attempts made to fill with gold. The patient returned with a phosphate filling. Examination showed it useless to apply the rubber. The cavity was prepared and filled with amalgam to the cervical border, and the remainder with gutta-percha, and the patient dismissed. At the next sitting, the amalgam was cut down for a fastener, the rubber applied and the tooth filled with gold. One year's time shows no discoloration from the amalgam.

No. 5.—Cavities upon buccal or labial surfaces, extending beneath the gums, so as to make it difficult to apply the rubber, especially the shallow crescent-shaped cavities in centrals and laterals. Prepare the cavity with no more depth than its necessary to remove softened dentine. Let the margin be distinct and the walls at right angles with the bottom of the cavity. Protect the teeth with napkin or paper, and fill with bibulous paper between the teeth on either side of the cavity. Dry the cavity and exposed gums thoroughly, and varnish both cavity and gums, allowing the varnish to pass beneath the gums where there is space, which will prevent moisture from capillary attraction. Line the cavity with crystal gold. First go around the border of the cavity, then build across until the cavity is lined with a basket of gold. Upon this foundation may be anchored a solid gold plug of any cohesive gold, without danger of falling out or decay around it.

This method is the same as that given by Dr. Howard in a clinic at Rochester, and it more than meets his claims in filling the porous dentine, whereby fluids are excluded and secondary decay prevented. With a little practice, filling this class of cavities is made easy.

Modification and adaptation of the principles here laid down will meet all conditions of anchorage for gold fillings, nor is the preparation of foundations less valuable for amalgam, gutta-percha, or phosphate fillings. The indications for use of varnish under oxy-phosphate, are sensitive dentine or near approach to the pulp. No acid excitement is experienced with this cavity lining.

Under gutta-percha it is valuable in fastening the filling to the walls of the cavity, in conditions unfavorable for the exclusion of moisture, as each piece of filling remains fixed in the cavity. It is true that chloro-percha, and some of the essential oils also, cause the filling to adhere.

but the lining is much softer and yielding than varnish. In connection with amalgam it may be used as with gold, as a lining for cavities where poorly calcified dentine must remain as the pulp covering; also in cases where dependence is placed upon oxydation to fill the dentine. So far as I can determine, this answers the same purpose, with much better appearance. Amalgam fillings inserted in heavy varnish remain bright upon the surface in contact with the cavity walls, which is not the case in any amalgam touching dentine,

Again, where there is no action upon the metal there is none upon the dentine; thus amalgam which contains no copper may be used with equally good results, without discoloring the teeth.

In conclusion, science teaches that the result of every operation in filling is the outcome of principles laid in the foundation and observed to completion. Whatever is success at any time will be for all time, under the same circumstances and conditions.

Progress is based upon the knowledge and application of principles leading to better methods. Thus we are stimulated to search for more knowledge and higher attainments.—*Dental Pract. and Advertiser.*

---

ARTICLE IV.

